# Immunohistochemical analysis of sensory corpuscles in human transplants of the anterior cruciate ligament

**DOI:** 10.1186/s13018-020-01785-5

**Published:** 2020-07-17

**Authors:** D. Rebmann, H. O. Mayr, H. Schmal, S. Hernandez Latorre, A. Bernstein

**Affiliations:** 1 Department of Orthopedics and Trauma Surgery, Medical Center - Albert-Ludwigs-University of Freiburg, Faculty of Medicine, Albert-Ludwigs-University of Freiburg, Hugstetter Straße 55, 79106 Freiburg, Germany; 2 Department of Knee, Hip and Shoulder Surgery, Schoen Clinic Munich Harlaching, Harlachinger Strasse 51, 81547 Munich, Germany

**Keywords:** Anterior cruciate ligament reconstruction, Proprioception, Immunohistochemistry, Mechanoreceptors

## Abstract

**Background:**

Sensory nerve endings in ligaments play an important role for the proprioceptive function. Clinical trials show that the sense of body position does not fully recover in the knee joint after reconstructive surgery of the ruptured anterior cruciate ligament. The aim of this study is to identify sensory corpuscles in autogenous and allogenous transplants of the ligament and to compare their quantity between the used allografts and autografts.

**Methods:**

Thirty-three patients were included in this study. Three patellar tendon allografts, 14 patellar tendon autografts and 12 semitendinosus autografts were harvested during revision surgery after traumatic rerupture of the graft. The control consisted of 4 healthy anterior cruciate ligaments after fresh rupture. After haematoxylin staining, immunohistochemical analysis was performed using antibodies against S100, p75 and PGP9.5. Microscopical examination was carried out, and the number of mechanoreceptors was counted. Statistical analysis was performed using the Mann-Whitney *U* test.

**Results:**

Two types of mechanoreceptors were identified in each graft: Ruffini corpuscles and free nerve endings. The number of Ruffini corpuscles per square centimeter was the highest in the control. Comparing the grafts, the highest number of receptors could be detected in the semitendinosus autograft. The amount of free nerve endings was higher in the semitendinosus and patellar tendon autografts than in the control; the allografts showed the lowest number of receptors. With increasing time after reconstruction, the number of both types of receptors showed a decrease in the semitendinosus graft, whereas it increased in the patellar tendon graft and allograft. The number of mechanoreceptors in the semitendinosus and patellar tendon graft decreased over time after graft-failure, whereas it increased slightly in the allograft.

**Conclusion:**

This study was the first to identify mechanoreceptors in human transplants of the anterior cruciate ligament. The partial increase in the number of receptors over time after reconstruction could indicate a reinnervation of the grafts.

## Introduction

The rupture of the anterior cruciate ligament mainly affects young, physically active people and is the most common injury of the knee [1]. To regain full function of the joint reconstruction of the ligament is the therapy of choice [2]. Currently, the most popular procedure is the use of a free, multi-stranded semitendinosus transplant [3]. An alternative method is the replacement with a quadriceps tendon graft or a patellar tendon graft which is removed with bone parts from patella and tibia [4]. Allografts from Achilles, quadriceps or patellar tendon are rarely used today due to risk of infection and uncertainty regarding long-term survival [5].

In a meta-analysis, Kim et al. showed that the proprioceptive function in the knee joint with ruptured anterior cruciate ligament is worse than in the healthy contralateral knee [6]. After reconstructive surgery, proprioception approximates the original state, as multiple clinical trials have shown [7–9], but it remains to be clarified whether this improvement is due to reinnervation of the graft or to re-establishment of proprioception by peri-articular structures. For this reason, the investigation of mechanoreceptors in transplants of ligaments is of great importance. The presence of these receptors is a necessity for proprioceptive sensation. At the time of this study, there were only few histological investigations of human cruciate ligament grafts. Young et al. carried out an immunohistochemical investigation of grafts regarding their neuronal structure in 2016. They took samples from ten patients who required either revision surgery or knee arthroscopy due to a different pathology. Four bone-tendon-bone patellar (BTBP) autografts, five BPTB allografts, one hamstring autograft and one healthy anterior cruciate ligament were stained with a monoclonal antibody against neurofilament protein (NFP) and then examined under a light microscope for positive structures. The authors described significantly fewer neuronal structures in the grafts compared to the control, whereas no significant difference could be observed within the graft groups [10]. Kim et al. found no mechanoreceptors in the examination of eleven Achilles tendon allografts, which they attributed to the fact that there is no significant reinnervation in the transplants [11].

In summary, there is no consensus concerning the presence of mechanoreceptors in transplants of the anterior cruciate ligament. Furthermore, it remains unclear whether there could be a reinnervation process after reconstruction of the ligament. For this reason, the aim of this study was to morphologically identify mechanoreceptors in the examined grafts and to correlate them with clinical parameters to obtain evidence of a possible reinnervation.

## Materials and methods

### Patients

Thirty-three samples were taken between 2007 and 2012 during revision surgery after graft failure or initial surgery for reconstruction of the anterior cruciate ligament. In two cases, samples were harvested during implantation of a total knee replacement. The mean age of the patients (23 men and 10 women) at the time of surgery was 31 years (range 17–74 years). Transplant groups were subdivided according to the length of stay and time from rerupture to revision surgery. The length of stay describes the time from implantation to rerupture of the graft. Exact numbers are shown in Table 1.

**Table 1:** Number of specimens in the control group and the graft groups. Graft groups are further divided in length of stay and time from rerupture to revision surgery

Transplant	Total number	Length of stay	Time from rerupture to revision
Control group	4		
Patellar tendon allograft	3	Unknown, 0	Unknown, 0
		6–12 months, 0	< 100 days, 2
		25–60 months, 1	> 100 days, 1
		61–120 months, 2	
		> 120 months, 0	
Patellar tendon autograft	14	Unknown, 0	Unknown, 0
		6–12 months, 0	< 100 days, 6
		25–60 months, 3	> 100 days, 8
		61–120 months, 8	
		> 120 months, 3	
Semitendinosus autograft	12	Unknown, 0	Unknown, 2
		6–12 months, 4	< 100 days, 6
		25–60 months, 4	> 100 days, 4
		61–120 months, 4	
		> 120 months, 0	

### Immunohistochemical staining

**Fig. 1:**
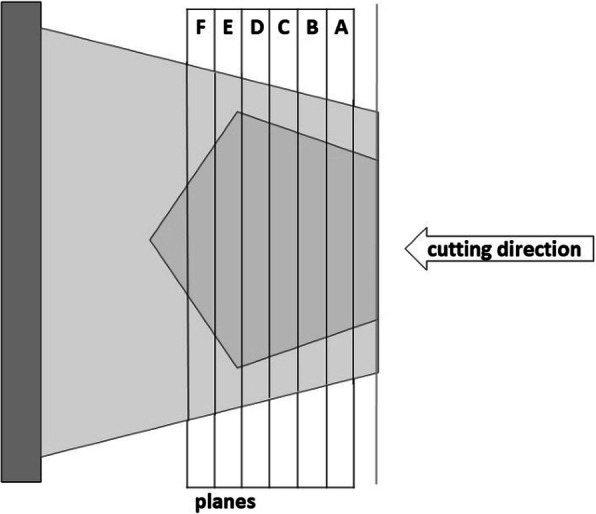
Sketch of the sectional planes A–F of one specimen. One plane equals 70 μm

Immediately after harvesting the specimens were fixed in 4% buffered formalin solution and embedded in paraffin. The number of sections to be made in each plane of the specimens was based on the size of the mechanoreceptors: Since a single receptor can extend over some 7 μm cuts, the distance between the cut planes was set to 70 μm. This procedure allows the number of receptors counted more than once to be kept very low [12]. One plane therefore consists of 10 sections; a total of 6 planes (A–F) were cut per sample as shown in Fig. 1.

In each of the planes, the first specimen was stained with HE, the second one with polyclonal antibody against S100 (abcam, ab15520), the third one with polyclonal antibody against p75 (sigmaaldrich, N3908) and the fourth one with monoclonal antibody against PGP9.5 (abcam, ab8189). These three antibodies are widely used in immunohistochemical analysis of neuronal structures and have proven to be the most reliant method in the detection of mechanoreceptors [11, 13–15].

### Antibodies

The S100-protein is expressed in myelinated and non-myelinated Schwann cells of the peripheral nervous system and in sensory neuronal structures such as mechanoreceptors [16]. It plays an important role in the intracellular calcium homeostasis; the organization of the cytoskeleton and some of the S100 proteins are segregated and fulfil extracellular functions which are similar to cytokines [17, 18].

The p75-receptor is part of the TNF-receptor family [19]. Like the others of these receptors, it induces apoptosis of the cell. Furthermore, there are other functions of the receptor which are subject of current research such as migration of Schwann cells, regulation of sensory neurons and myelinisation [20]. The p75-receptor is expressed both in myelinated and non-myelinated neurons [21].

Protein-Gene-Product 9.5 is a ubiquitin-carboxyl-hydroxylase, also known as UCH-L1 [22, 23]. Although it does not only occur in neuronal tissue, its concentration is up to 50 times higher in neurons. On the one hand, it is responsible for ubiquitination which marks damaged proteins and leads to their degradation [24]. On the other hand, it is assumed to play an important role in differentiation and growth of neuronal cells and synapses [25].

The slides were deparaffinized with xylene (3 × 10 min) and hydrated through incubation in an increasing alcohol sequence (100 °C, 2 × 96 °C, 70 °C each for 10 min, H_2_O dest. for 5 min). Subsequently, the antigens had to be demasked for the anti-p75 and anti-PGP9.5 staining by incubation in citrate buffer at 95 °C for 40 min. Afterwards, each specimen was incubated with 3% H_2_O_2_ for 10 min to block endogenous peroxidase. Blocking solution then was applied to the slides for 5 min before they were incubated with the primary antibody overnight in a wet chamber at 4 °C (dilutions: anti-S100: 1/100, anti-p75: 1/200, anti-PGP9.5: 1/250). Human malignant melanoma was used as positive control for the anti-S100 antibody, neonatal brain of the rat for the anti-p75 antibody and neonatal pancreas of the rat for the anti-PGP9.5 antibody. After removal of the antibodies, postblocking solution was applied for 20 min, before the slides were incubated with the secondary antibody (horseradish-peroxidase polymer, HRP) for 30 min. Between each step, the slides were rinsed with phosphate-buffered saline (PBS) 3 times. Following, they were developed in aminoethyl carbazole (AEC) for 10 min (anti-S100), respectively, and 15 min (anti-p75 and anti-PGP9.5). The reaction between the HRP and AEC was then stopped with H_2_O dest., and counterstaining with Mayer’s HTX (5 min) was performed. After rinsing with tap water for 10 min, the slides were fixed with aqueous mounting medium.

### Histological examination

The histological examination of the stained sections was performed by the first author (D.R.); randomized samples were examined by a second investigator (A.B.). First, overview images were taken using an Olympus BX53 light microscope to measure the area of the sections. Mechanoreceptors were analysed according to the classification of Freeman and Wyke. This classification consists of four types of receptors: I, Ruffini-endings; II, Pacinian-corpuscles; III, Golgi-like endings; and IV, free nerve endings [26]. Mechanoreceptors were counted through an Olympus BX 51 light microscope in every slide stained with immunohistochemistry, which means three slides per section in six sections per specimen. The number of receptors was then stated per square centimeter. Due to the high reliability, the number counted in the sections stained with the anti-S100 antibody was used to determine the number of receptors per square centimeter [12, 16]. Sections stained with the other antibodies were used to clearly identify each receptor.

Data are given as mean ± standard deviation. The IBM SPSS statistics program (version 24, IBM Corporation) was used for statistical evaluation. Since the results were not normally distributed, data were analysed using the Mann-Whitney *U* test to compare the independent samples. A *p* < 0.05 was considered statistically significant. The number of mechanoreceptors per square centimeter compared to the native ACL was set as the primary endpoint.

## Results

When the samples were taken, important information about the clinical status of the joint and accompanying injuries could be collected intraoperatively. To put the results shown below into a clinical context, these findings as well as the age of the patients are presented in Table 2.

**Table 2:** Clinical data provided by the surgeon (H.O.M.), obtained through intraoperative examination of the joint and mean age of the patients with range in years

		CG	STG	PTG	AG
Gonarthrosis	None	4	5	5	1
	Initial stadium	0	7	5	2
	Manifest arthrosis	0	0	4	0
Cartilage lesion	Not given	0	1	1	0
	None	3	0	0	0
	Superficial lesion	1	8	7	2
	Deep lesion	0	3	6	1
Medial meniscus	Intact	3	6	8	3
	Contusion/compression/fraying	1	6	6	0
Accompanying lesions	None	3	11	13	3
	Rupture of MCL	1	1	0	0
	Tibial plateau fracture	0	0	1	1
Mean age of patients in years		31 (17–48)	27 (20–52)	39 (20–74)	39 (37–41)

*MCL* medial collateral ligament, *CG* control group

**Fig. 2:**
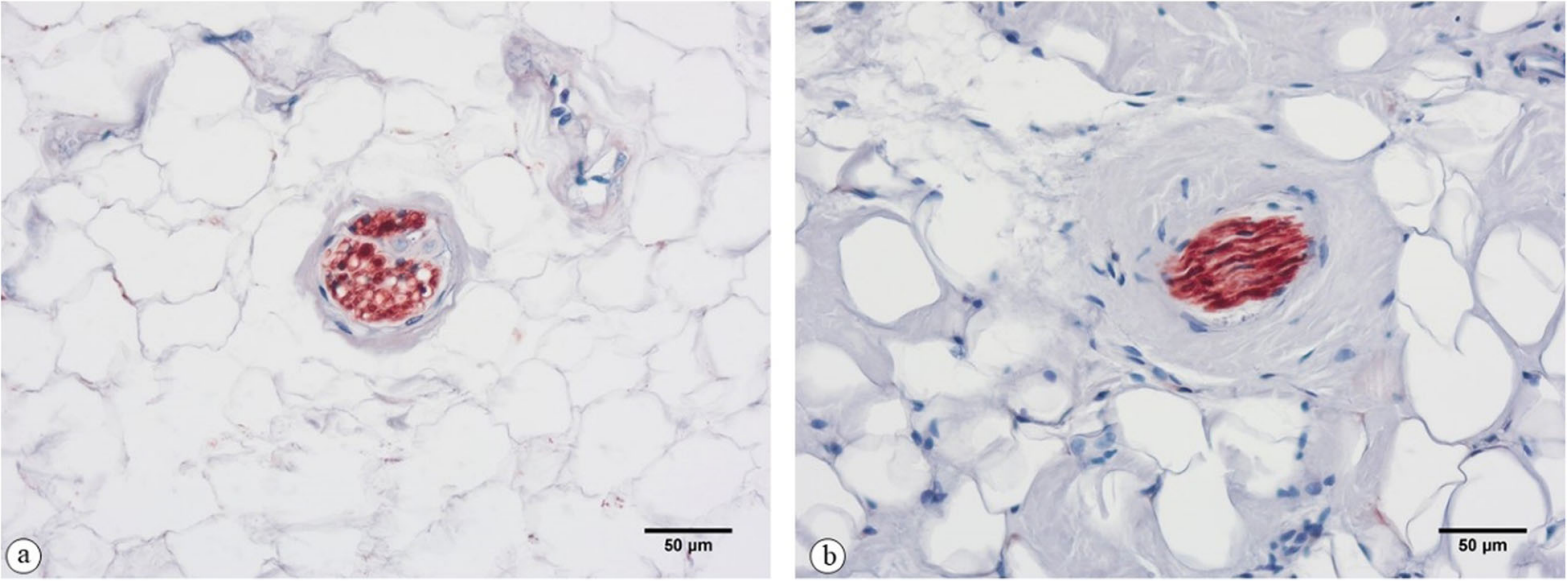
Ruffini ending (**a**) and free nerve ending (**b**). Staining with the anti-S100 antibody

Two types of mechanoreceptors could be detected in each of the investigated graft groups and the control group: Ruffini endings (type I) and free nerve endings (type IV). Pictures of these receptors stained with the anti-S100 antibody are shown in Fig. 2. Pacini corpuscles or Golgi endings could not be detected in any of the slides. Pictures of these receptors stained with the anti-PGP9.5 and anti-p75 antibody can be found in the additional files (additional files 1 and 2).

**Fig. 3:**
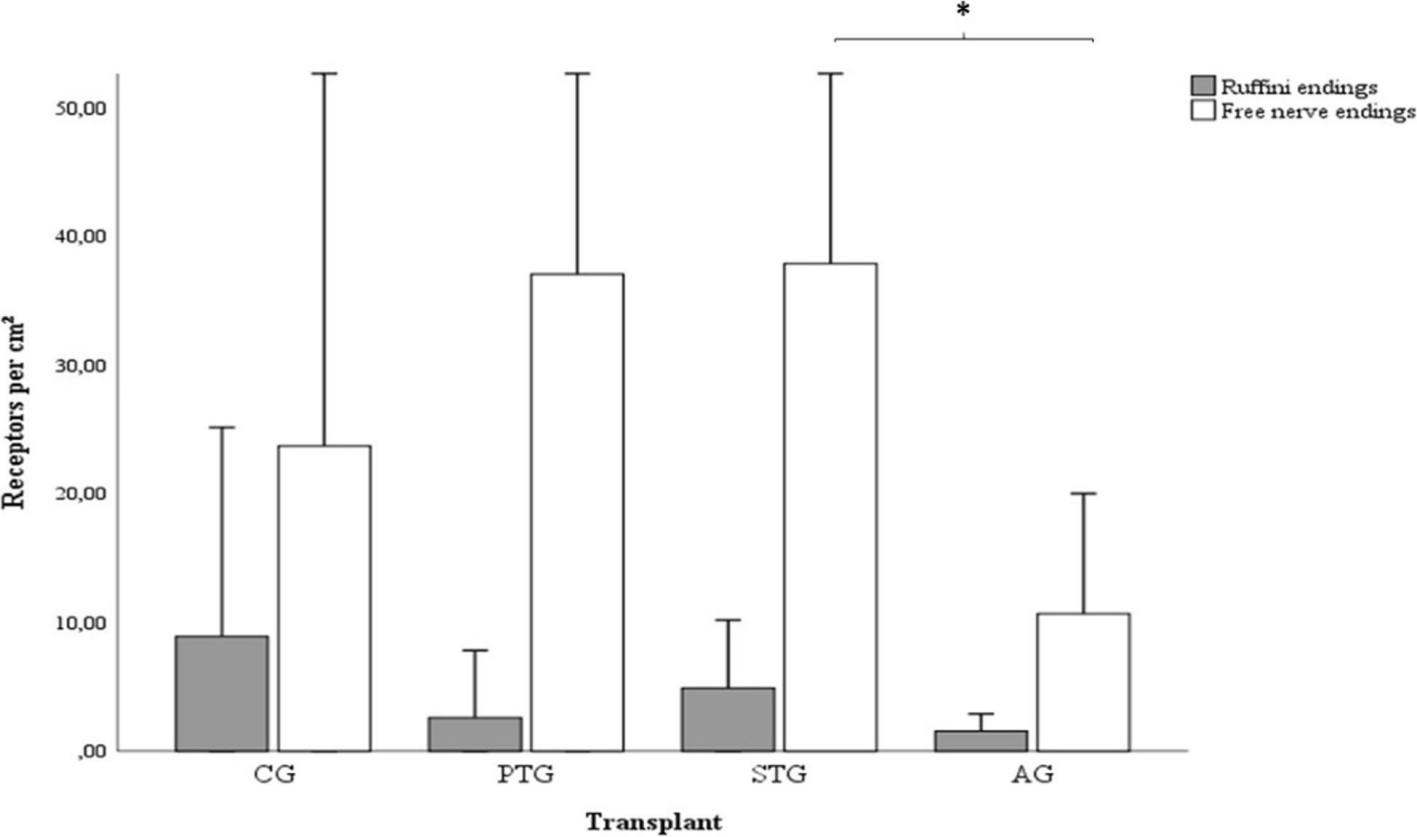
Distribution of mechanoreceptors in the control group (CG) and the graft groups (AG, patellar tendon allograft; STG, semitendinosus autograft; PTG, patellar tendon autograft) determined with the anti-S100 antibody. Asterisk (*) indicates significant difference in the number of free nerve endings between AG and STG (*p* = 0.048)

The control showed 8.88 (± 16.27) Ruffini endings and 23.69 (± 31.97) free nerve endings per square centimeter. With 4.88 (± 5.27) type I receptors and 37.87 (± 27.90) type IV receptors per square centimeter, the number of mechanoreceptors was the highest in the investigated semitendinosus autografts. The patellar tendon autografts showed a lower number of receptors with 2.56 (± 5.24) Ruffini endings and 37.03 (± 28.02) free nerve endings per square centimeter. The lowest number of receptors was detected in the three investigated patellar tendon allografts with 1.53 (± 1.34) type I receptors and 10.65 (± 9.32) type IV receptors per square centimeter (Fig. 3).

The difference in the number of free nerve endings between the patellar tendon allografts and the semitendinosus autografts showed a statistical significance (*p* = 0.048).

To gain insight into a possible reinnervation of the transplants, the graft groups were subdivided according to the length of their stay in the joint. The periods of time in which the groups were divided as well as the number of receptors found in each group are shown in Table 3.

**Table 3:** Number of specimens per time period (length of stay between implantation and rerupture) and numbers for Ruffini endings and free nerve endings per square centimeter for each time period

		6–12 months	25–60 months	61–120 months	> 120 months
STG	Number of specimens	4	4	4	–
	Ruffini endings/cm^2^	7.03 ± 7.98	2.71 ± 3.56	4.88 ± 3.63	
	Free nerve endings/cm^2^	55.10 ± 35.36	32.78 ± 29.56	25.72 ± 10.15	
PTG	Number of specimens	–	3	8	3
	Ruffini endings/cm^2^		0.74 ± 1.27	1.89 ± 3.18	6.19 ± 10.72
	Free nerve endings/cm^2^		20.38 ± 11.57	33.01 ± 33.93	64.40 ± 60.22
AG	Number of specimens	–	1	2	–

*STG* semitendinosus autograft, *PTG* patellar tendon autograft, *AG* patellar tendon allograft

With increasing time from implantation, the mechanoreceptors especially in the patellar tendon autografts showed an increase in their number for both Ruffini endings (0.74 ± 1.27 ➔ 1.89 ± 3.18 ➔ 6.19 ± 10.72 per cm^2^) and free nerve endings (20.38 ± 11.57 ➔ 33.01 ± 33.93 ➔ 64.40 ± 60.22 per cm^2^). The semitendinosus grafts showed a decreasing number of free nerve endings after 120 months (55.10 ± 35.36 ➔ 32.78 ± 29.56 ➔ 25.72 ± 10.15 per cm^2^) and an inconclusive development in the number of Ruffini endings (7.03 ± 7.98 ➔ 2.71 ± 3.56 ➔ 4.88 ± 3.63 cm^2^). Since the number of samples for the allografts was only three, there were no reliable results.

Furthermore, the number of receptors could be analysed concerning the time from rerupture of the graft until revision surgery. Two groups were set up for this purpose: grafts which stayed less than 100 days in the joint after rerupture and grafts which stayed longer than 100 days. The number of specimens and mechanoreceptors per group is shown in Table 4. For two patients with a semitendinosus graft information about this period could not be obtained.

**Table 4:** Number of specimens per time period between rerupture and revision surgery and numbers for Ruffini endings and free nerve endings per square centimeter for each time period

		< 100 days	> 100 days
STG	Number of specimens	6	4
	Ruffini endings/cm^2^	6.43 ± 6.41	2.50 ± 2.15
	Free nerve endings/cm^2^	42.38 ± 34.14	41.09 ± 23.77
PTG	Number of specimens	6	8
	Ruffini endings/cm^2^	4.92 ± 7.62	0.80 ± 1.13
	Free nerve endings/cm^2^	64.89 ± 44.64^a^	16.13 ± 9.79^b^
AG	Number of specimens	2	1

Letters a and b show statistically significant differences (*p* = 0.039)

Generally, it has been shown that the number of receptors decreases with increasing time from rerupture. In the investigated semitendinosus grafts, the number of receptors in the second group was less than half compared to the first one (6.43 ± 6.41 ➔ 2.50 ± 2.15 per cm^2^) whereas the number of free nerve endings only showed a slight decrease (42.38 ± 34.14 ➔ 41.09 ± 23.77 per cm^2^). These findings were even more evident in the patellar tendon autografts, where both Ruffini endings (4.92 ± 7.62 ➔ 0.80 ± 1.13 per cm^2^) and free nerve endings (64.89 ± 44.64 ➔ 16.13 ± 9.79 per cm^2^) showed a clear decrease. The development for the free nerve endings showed a statistical significance (*p* = 0.039). Again, no reliable results for the allografts could be obtained due to the low number of specimens.

## Discussion

For the first time, this study could prove the existence of mechanoreceptors in human transplants of the anterior cruciate ligament. Ruffini endings and free nerve endings could be identified in every graft group. The previously mentioned study of Young et al. also found neuronal structures both in patellar tendon allo- and autograft and in hamstring autografts. The authors described a significantly lower number of NFP-positive structure in the transplant groups compared to the control group. They did not find a difference between the transplant groups [10]. In this study, the number of Ruffini endings was also lower in the graft groups, but the difference to the control showed no statistical significance.

Multiple studies on animals have already shown mechanoreceptors in transplants of the anterior cruciate ligament: Barrack et al. not only found types I, II and IV receptors in patellar tendon autografts in dogs but also derived sensory evoked potentials (SEP) by stimulating the grafts [27]. Samples taken as biopsies from human Achilles tendon allografts and stained with the anti-S100 antibody on the other hand did not show any sensory nerve endings [6, 15]. An explanation for the different results could be the removal technique and the different original tissues.

The increasing number of receptors in the patellar tendon grafts with a longer time of stay in the joint could be a sign of reinnervation. These findings also were previously shown in animal experiments. Fromm and Kummer could prove that ACL allografts in canines show significantly more nerve endings 24 and 36 weeks after implantation than after 12 weeks [28]. Wada et al. compared the number of Ruffini endings in patellar tendon autografts in canines with the number of receptors in the contralateral anterior cruciate ligament. Twenty weeks after implantation, there was no statistical significance between the control group and the transplant group. The authors considered this as a sign for a reinnervation of the grafts [29]. Further studies on human transplants are needed to prove the reinnervation of the transplants.

In the present study, the development of the receptors after rupture of the grafts could be described for the first time; there are no data in the literature describing this process. The number of sensory corpuscles in the remnants of the ruptured ACL on the other hand has already been extensively investigated: a meta-analysis by Kosy et al. from 2017 describes that the decrease in mechanoreceptors with increasing time from rupture could be demonstrated in multiple histological studies [30]. A microscopic examination of the ruptured ACL from Murray et al. showed an increase of blood vessels and cell count 8 to 20 weeks after rupture [31]. Thus, the decrease of sensory corpuscles after rupture of the grafts presented in this study seems to be similar to the one in remnants of the ruptured ACL. There is no evidence that the development of nerve endings corresponds to the increase of blood vessels and cell count.

To compare the number of sensory nerve endings between the grafts and the original tendons (patellar tendon and semitendinosus tendon), data from the literature was used. Çabuk et al. investigated all ligaments of the human knee concerning mechanoreceptors by using an antibody against S100 [32]. Table 5 shows the numbers for the original tissue as found by the authors compared with the data for the grafts as obtained in this study.

**Table 5:** Numbers of mechanoreceptors per square centimeter in the graft groups, the control group and the original tendons. Staining with the anti-S100 antibody

Tissue	Ruffini endings/cm^2^	Free nerve endings/cm^2^
Anterior cruciate ligament	8.88 ± 16.27	23.69 ± 41.97
Semitendinosus autograft	4.88 ± 5.27	37.87 ± 27.90
Semitendinosus tendon*	0.24 ± 0.34	7.14 ± 11.14
Patellar tendon autograft	2.56 ± 5.24	37.03 ± 38.02
Patellar tendon allograft	1.53 ± 1.34	10.65 ± 9.32
Patellar tendon*	1.00 ± 1.78	10.74 ± 11.12

Staining with the anti-S100 antibody

*Data from Çabuk et al.

In general, the number of mechanoreceptors is higher in all the investigated grafts compared to the original tendons. This difference becomes particularly clear for the semitendinosus graft, but even the patellar tendon allograft showed more Ruffini endings than the native patellar tendon. Furthermore, the results for the graft groups are closer to the control than to the ones for the original tendons. These results are not statistically significant, so they can only be regarded as a tendency. A meta-analysis by Pauzenberger et al. concerning the remodelling of ACL grafts showed that multiple studies on animals and humans could prove an approximation in the structure of the transplants towards the ACL [33]. The investigated parameters included cellularity, vascularity, myofibroblast density and collagen orientation [34]. Numbers for the mechanoreceptors in the transplants as shown in Table 5 could be a sign of remodelling also in regard of sensory nerve endings. A clinical trial by Ozenci et al. could prove significantly better proprioception after reconstruction of the ACL in comparison to the injured ACL using the threshold to detect passive motion test (TDPM). The authors did not find any difference between autograft and allograft [9]. The results of the present study indicate a superiority of autografts over allografts concerning the number of mechanoreceptors, which could become an important factor in graft choice. In order to place these findings in a clinical context, it is necessary to consider the status of the examined joints and the accompanying injuries, which occurred during the traumatic rupture. Exact numbers are shown in Table 2, which is why only the most important insights should be stated here. More than half of the joints in the transplant groups showed at least signs of initial gonarthrosis (STG, 58%; PTG, 64%; AG, 67%), whereas the controls did not show any sign of arthrosis. The risk of developing arthrosis after rupture and reconstruction of the ACL is a known fact investigated in many studies [35, 36], which could be an explanation for the occurrence of osteoarthritis in the transplant groups. Furthermore, cartilage lesions were found in nearly every joint with an ACL transplant; a damage of the medial meniscus could be described in nearly half of the cases after rupture of the semitendinosus or patellar tendon autograft. In contrast, the control group showed mostly no cartilage damage. Accompanying lesions such as rupture of the MCL or tibial plateau fracture were rarely seen.

## Limitations

There are some limitations to be considered looking at the results of this study. First, the population of which the samples were taken was inhomogeneous. For this reason, clinical parameters could not be correlated with the number of receptors. Furthermore, the control consisted of traumatically ruptured ACL; no healthy native tissue could be included in this study. Therefore, the anatomic localisation of receptors could not be shown.

## Conclusion

This study was the first to identify mechanoreceptors in human transplants of the anterior cruciate ligament. The partial increase in the number of receptors over time after reconstruction could indicate a reinnervation of the grafts. Two types of mechanoreceptors could be identified in the investigated allo- and autografts as well as the control group. There was a significant difference for the free nerve endings between semitendinosus autograft and patellar tendon allograft. Grafts with a longer length of stay showed more mechanoreceptors; this could indicate a possible reinnervation. After rerupture, the number of sensory nerve ending was decreasing with increasing time. The number of receptors in the grafts was higher than in the original tendons (patellar tendon and semitendinosus tendon), which could be a sign of remodelling.

## Supplementary information

**Additional file 1.** Ruffini ending (a) and free nerve ending (b). Staining with the anti-p75 antibody.

**Additional file 2.** Ruffini ending (a) and free nerve ending (b). Staining with the anti-PGP9.5 antibody.

## Data Availability

The datasets used and or analysed during the current study are available from the corresponding author on reasonable request.

## Abbreviations

ACL: Anterior cruciate ligament
AEC: Aminoethyl carbazole
BTBP: Bone-to-bone patellar autograft
dest.: Distilled
HE: Haematoxylin and eosin
HRP: Horseradish-peroxidase polymer
HTX: Haematoxylin
MCL: Medial collateral ligament
NFP: Neurofilament protein
PBS: Phosphate-buffered saline
SEP: Sensory evoked potentials
UCH-L1: Ubiquitin-carboxyl-hydroxylase
