# Supplementary figures and images for: Immunohistochemical analysis of sensory corpuscles in human transplants of the anterior cruciate ligament
Source: J Orthop Surg Res. 2020 Jul 17;15:270. doi: 10.1186/s13018-020-01785-5 (PMC7368668; doi:10.1186/s13018-020-01785-5)

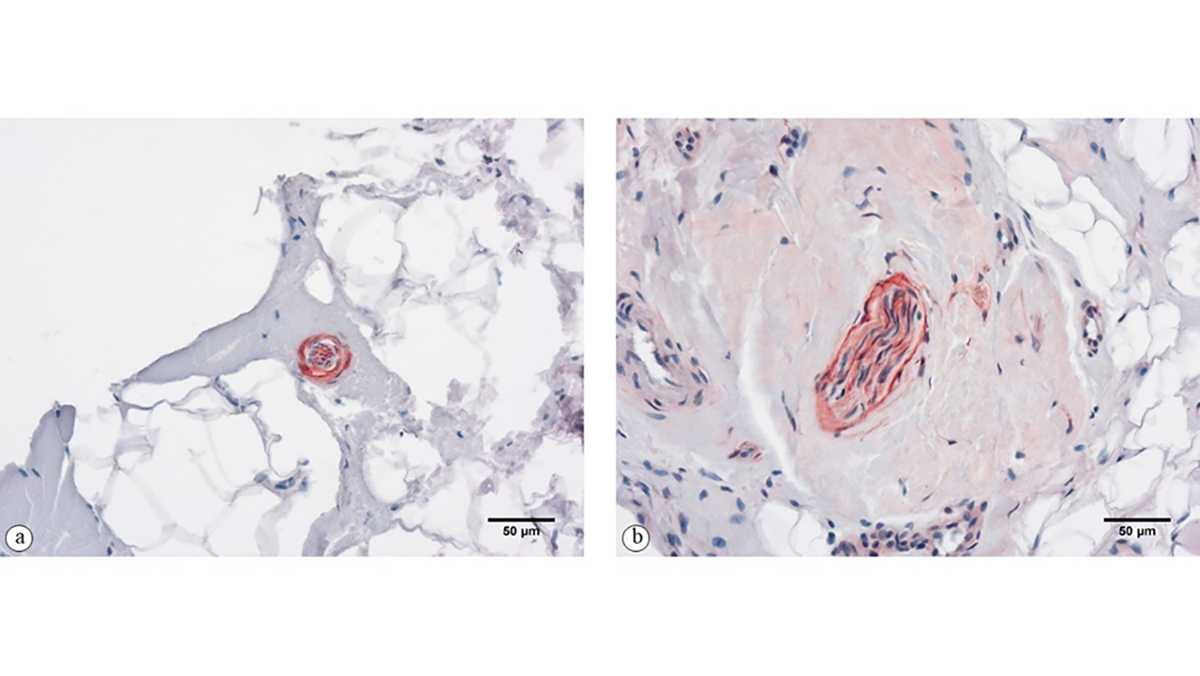

Supplement: Supplementary file 1 — Additional file 1. Ruffini ending (a) and free nerve ending (b). Staining with the anti-p75 antibody. [file 13018_2020_1785_MOESM1_ESM.tif]

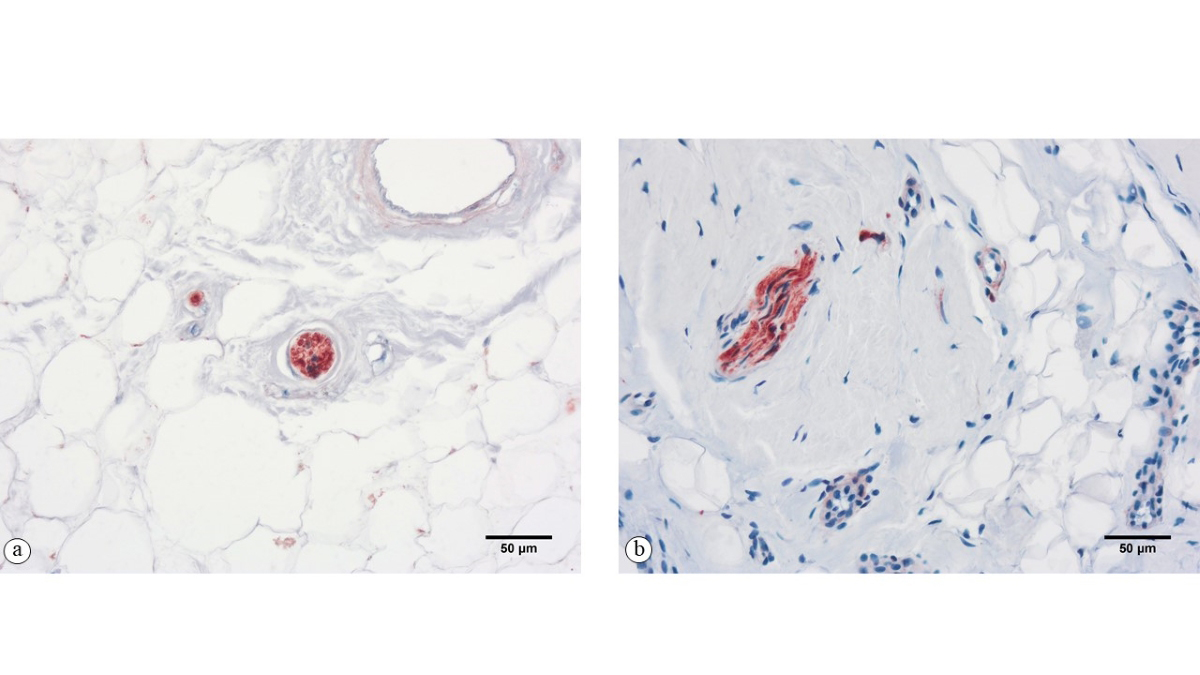

Supplement: Supplementary file 2 — Additional file 2. Ruffini ending (a) and free nerve ending (b). Staining with the anti-PGP9.5 antibody. [file 13018_2020_1785_MOESM2_ESM.tif]
